# Mechanism of PDZK1 in Hepatocellular Carcinoma Complicated with Hyperuricemia

**DOI:** 10.1155/2022/1403454

**Published:** 2022-11-14

**Authors:** Linqi Guo, Wenda Jiang, Lingli Quan, Xinli Teng, Jing Zhao, Hongbin Qiu

**Affiliations:** ^1^School of Basic Medicine Jiamusi University, Jiamusi 154000, China; ^2^Department of General Surgery, The First Affiliated Hospital of Jiamusi University, Jiamusi 154000, China; ^3^Pulmonary and Critical Care Medicine 1, The Affiliated Zhuzhou Hospital Xiangya Medical College CSU, Zhuzhou 412000, China; ^4^Medical Oncology, The Tumor Hospital of Jiamusi, Jiamusi 154000, China; ^5^School of Public Health Jiamusi University, Jiamusi 154000, China

## Abstract

**Background:**

Hepatocellular carcinoma (HCC) is a kind of primary liver cancer that accounts for more than 90% of primary hepatocellular carcinomas. Hyperuricemia is closely related to the development, recurrence, metastasis, and prognosis of cancer. Previous studies have proved that the serum uric acid level can increase the incidence rate and mortality of malignant tumors. However, the specific pathogenesis remains unstudied.

**Methods:**

RT-qPCR analysis showed that the mRNA expression of PDZK1 and ABCG2 increased significantly after HCC cells were exposed to different concentrations of soluble uric acid (2.5, 5, 10, 20 mg/dl) for 24 hours. Then, in HCC shRNAs, PDZK1, or over expression PDZK1 were used. CCK8, wound healing, and Transwell assay showed that PDZK1 regulates cell proliferation, invasion, and migration. Flow cytometry results revealed that PDZK1 affects cell apoptosis. Western blot results show that PDZK1 affects the STAT3/C-myc pathway. Then, in vivo tumorigenesis, allopurinol maybe an effective drug to advance: the prognosis of HCC.

**Results:**

In our study, RT-qPCR analysis showed that the mRNA expression of PDZK1 and ABCG2 increased significantly after different concentrations of soluble uric acid in HCC. Then, PDZK1 affects the proliferation, migration, and apoptosis of HCC through the STAT3/C-myc pathway.

**Conclusions:**

Hyperuricemia response affects the expression of PDZK1; PDZK1 affects the proliferation, migration, and apoptosis through the STAT3/C-myc pathway in hepatocellular carcinoma. It is suggested that PDZK1 maybe closely related to the occurrence, development, and prognosis of HCC and allopurinol maybe have potential anticancer effects.

## 1. Introduction

Hepatocellular carcinoma (HCC) is a type of primary liver cancer that accounts for more than 90% of primary hepatocellular carcinomas. HCC is currently the fifth most common cause of cancer in the world [1]. The second-leading cause of cancer death in men is HCC. The five-year survival rate of HCC is about 18%, just after pancreatic cancer [2, 3]. Important risk factors for hepatocellular carcinoma include viral hepatitis B or viral hepatitis C, alcoholic liver disease, nonalcoholic fatty liver disease, and so on [4, 5]. Approximately 80%–90% of patients with cirrhosis develop liver cancer [6]. Therefore, it is very important to explore the occurrence, development, and potential molecular mechanism of liver cancer.

A product of purine oxidative metabolism is uric acid (UA). Some studies point out that the proportion of tumor patients with ranges hyperuricemia from high to low for vocal cord cancer, maxillary cancer, hypopharyngeal cancer, bladder cancer, liver cancer, and ovarian cancer [7–10]. Because of its proinflammatory characteristics, hyperuricemia may have an important role in the pathogenesis of cancer. Its mechanism may be related to reactive oxygen species (ROS), inflammatory corpuscle activation, and xanthine oxidoreductase (XO) mediated production of active free radicals [11–15]. It is closely related to the incidence, mortality, and prognosis of many solid tumors.

Allopurinol is the first-line medicine to treat hyperuricemia. Combining allopurinol with other medicines had been extensively explored. Previous clinical trials have shown that allopurinol has a positive association with prostate cancers, patients treated with allopurinol could decreased the incidence of prostate cancer. And the combined use of allopurinol for one month can reduce the level of NF kappaB in patients with colonic adenoma [16–19]. It is suggested that the drug that reduces uric acid levels have potential anticancer effects and provides a new idea for tumor treatment.

PDZ domain-containing 1 (PDZK1) is located on chromosome 1 q21.1. It has a relative molecular mass of 63 KDa and contains 519 amino acids. It contains 4 protein domains; the PDK domain of PDZK1 is mainly involved in regulating the subcellular localization of various uric acid transporters, and some studies have found that its rs12129861 mutation is bound up with hyperuricemia and gout pathogenesis [20–22]. In recent years, studies have found that the abnormal expression level of PDZK1 was found in various tumors, for example breast cancer, renal cell carcinoma, and gastric cancer [23–25]. However, the molecular mechanism of PDZK1 in HCC remains unclear.

In our study, the mRNA expression of PDZK1 was significantly increased after HCC after different concentrations of soluble uric acid treatment, and PDZK1 affects the proliferation, migration, and apoptosis of HCC through the STAT3/C-myc pathway. PDZK1 maybe closely related to the occurrence, development, and prognosis of HCC and allopurinol may have potential anticancer effects.

## 2. Materials and Methods

### 2.1. Cell Culture and Construction Lentivirus Vectors

HepG2 and Hep3B cells were acquired from ATCC, Huh7, and PVTT cells were acquired from the Institute of Cell Biochemistry, Chinese Academy of Sciences. The cells were incubated in RPMI-1640 (Gibco, CA, USA) medium containing 10% FBS (Invitrogen, CA, USA) and cultured at 37°C and 5%CO_2_ incubator. sh-PDZK1 (SH1, SH2) and sh-control (SC) were constructed by ribobio Biotechnology Co., Ltd (GuangZhou China). The overexpression vector of PDZK1 (pcDNA-PDZK1, PDZK1) and control lntivirus vectors (pcDNA-NC, vector) were constructed by GenePharma Biotechnolog Co., Ltd (Suzhou, China). The cells were transfected by using Lipofectamine™ 3000 (Invitrogen, CA, USA) followed by the protocols of the manufacturer.

### 2.2. CCK8 Analysis

HepG2, Hep3B cells (2 × 10^3^) or Huh7, and PVTT cells (4 × 10^3^) were incubated in a 96-hole plate after 48 hours of transfection, after 24 hours, 48 hours, and 72 hours. CCK8 reagent (10 *μ*L) was added into each hole for 2 h, and each group repeated 3 times. The absorbance of each hole was measured by a microplate assay (EnSpire 2300, PerkinElmer, USA) at a wavelength of 450 nm.

### 2.3. Wound Healing

HepG2, Hep3B cells (5 × 10^5^) or Huh7, and PVTT cells (8 × 10^5^) were incubated into the 6-hole plate after 48 hours of transfection, after 24 hours, a vertical line was drawn evenly in the middle of the hole base using a 20 *μ*L pipette tip, and each group was repeated 3 times. Images were taken at 0 and 24 hours under a light microscope to observe cell migration.

### 2.4. Transwell Assay

Cells (1 × 10^5^) were added to the Transwell upper chamber without fetal bovine serum after 48 hours of transfection. A culture medium (600 *μ*L) containing 15% fetal bovine serum was added into the lower chamber. After 48 hours, the noninvasive cells in the upper chamber were gently wiped away by a cotton swab. The lower chamber cells were stained with crystal violet. Then, observed and counted cells by inverted microscope. Each group was repeated 3 times.

### 2.5. Flow Cytometry Assay

5 × 10^7^ cells were collected in centrifuge tubes after 48 hours of transfection, then, PBS was washed twice. Cells were stained with 5–10 *μ*L propidium iodides (PI) and 5–10 *μ*L Annexin V-FITC into 100ul stain buffer, 15 mins later, cell apoptosis was detected by BD FACSCalibur (BD, USA), each group was repeated 3 times, and analyzed by Cell Quest software.

### 2.6. RNA Extraction and RT-PCR Analysis

Cells (5 × 10^6^) were collected after 48 hours of transfection, and total RNA was extracted by Trizol. 1 *μ*g RNA was used to synthesis of cDNA according to the instructions of the reverse transcription kit (Agilent, USA). The mRNA expression of PDZK1 was determined by RT-PCR on the CFX96Tm real-time System (Bio-Rad, USA), as follow: 95°C, 3 min, then, 95°C20 s and 70°C 1 min for 40 cycles. The calculation method of relative expression used the comparative Ct (2^−ΔΔCt^) method [26], and each group was repeated 3 times.

### 2.7. Western Blot Assay

Transfected HCC cells were collected after 48 hours, the culture medium was discarded, PBS was washed twice, and then, cell lysate was added. Following the manufacturer's protocol of the BCA Kit (Thermo Scientific, USA) to quantify total protein, then, the primary antibodies (STAT3, p-STAT3, C-myc, and GAPDH) (Cell Signaling Technology, USA) were incubated for 12 hours, and the second antibody (Cell Signaling Technology, USA) were added for 2 hours. Future, chemiluminescent reagent was added and incubated in the dark for 5 min, developed for 30 s, fixed for 10 s, and each group was repeated 3 times. The gray value of electrophoresis band of the protein was analyzed by Image J software.

### 2.8. In Vivo Tumorigenesis

Transfected HCC cells were collected after 48 hours. Then, digestion by trypsin, centrifuged, counted. The corresponding volume of cell suspension was measured by the subcutaneous injection of 5 × 10^6^ cells into mice with hyperuricemia. After subcutaneous transplantation of mice, allopurinol was fed every four days. After 4 weeks, the serum of the tail vein of nude mice was taken, the animals were killed, and the tumor tissue was taken. Measure the tumor size with a vernier caliper and calculate the tumor volume formula: volume = 0.5 *∗* length *∗* width *∗* width.

### 2.9. Statistical Analysis

SPSS 20 was used to analyze the experimental data. A Mean ± standard was used to represent data between groups. A one-way ANOVA test was used for statistical analysis, and a *t*-test was used for the comparison between two groups.

## 3. Results

### 3.1. The Expression of PDZK1 and ABCG2 in Hepatocellular Cells Is Mediated by the Stimulation of Soluble Uric Acid

**Figure 1:**
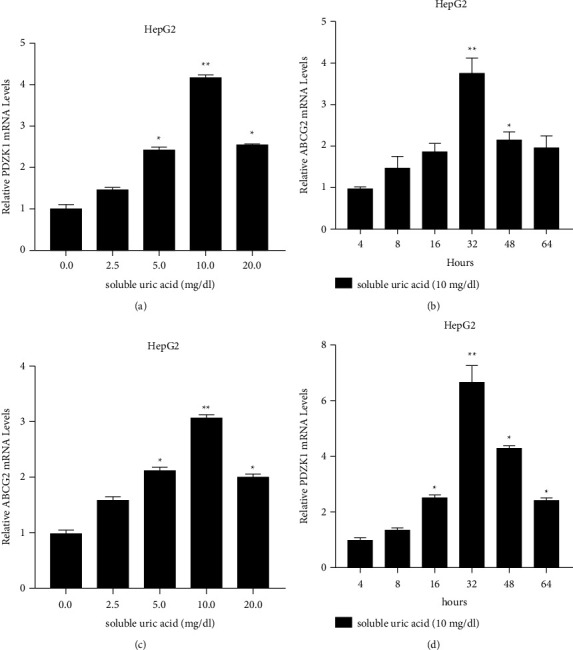
The expression of PDZK1 and ABCG2 in HCC is mediated by the stimulation of soluble uric acid. (a, b) The mRNA expression of PDZK1 and ABCG2 were significantly increased in hepatocellular cells were exposed to different concentrations of soluble uric acid (2.5, 5, 10, and 20 mg/dl) for 24 hours. (c, d) The mRNA expression of PDZK1 and ABCG2 after treated with 10 mg/dl soluble uric acid for 4, 8, 16, 32, 48, and 64 hours, data represent mean ± SD. ^*∗*^*P* < 0.05 compare with negative control.

RT-qPCR analysis showed that the mRNA expression of PDZK1 and ABCG2 increased significantly were exposed to different concentrations of soluble uric acid (2.5, 5, 10, and 20 mg/dl) in HCC cells (Figures 1(a) and 1(b)). After the cells were treated with 10 mg/dl soluble uric acid for 4, 8, 16, 32, 48, and 64 hours, the expression of PDZK1 and ABCG2 peaked at 32 hours (Figures 1(c) and 1(d)). It indicates that hyperuricemia response affects the expression changes of PDZK1 is more obvious, and then, we choose PDZK1 in the next study.

### 3.2. PDZK1 Affects the Proliferation, Migration, and Apoptosis of HCC

**Figure 2:**
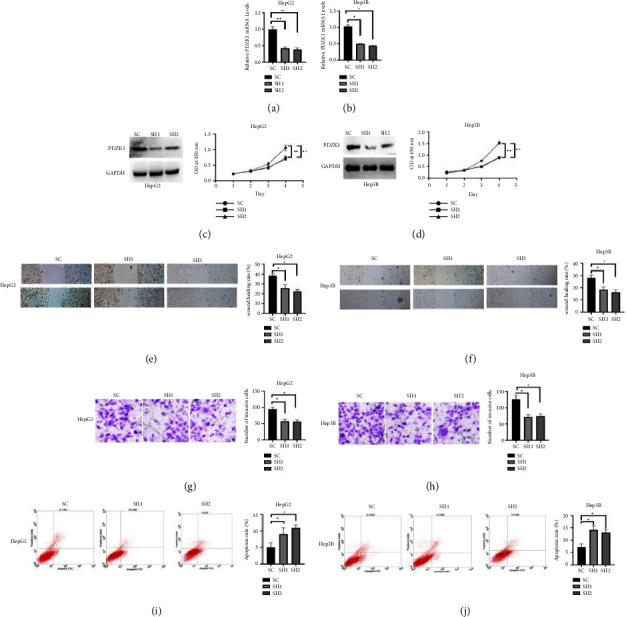
sh-PDZK1 inhibit the proliferation, migration, and promote apoptosis of HCC. (a, b, c, d) Western blot and RT-qPCR analysis showed that PDZK1 shRNAs strongly inhibited the expression of PDZK1, and the expression level decreased significantly. (e, f, g, h) CCK8, wound healing, and transwell assay showed that sh-PDZK1 induced the inhibition of cell proliferation, invasion, and migration. (i, j) Flow cytometry results revealed that sh-PDZK1 induced cell apoptosis. Data represent mean ± SD. ^*∗*^*P* < 0.05 compare with negative control.

We used shRNAs to knock down PDZK1 in HepG2 and Hep3B cells. The interfering shRNA was transfected into negative control cells. Western blot and RT-qPCR analysis showed that PDZK1 shRNAs strongly inhibited the expression of PDZK1, and the expression level decreased significantly (Figures 2(a)–2(d)). CCK8, wound healing, and transwell assay showed that sh-PDZK1 could inhibit ell proliferation, invasion and migration (Figures 2(e)–2(h)). Flow cytometry results revealed that sh-PDZK1 induced cell apoptosis (Figures 2(i) and 2(j)).

**Figure 3:**
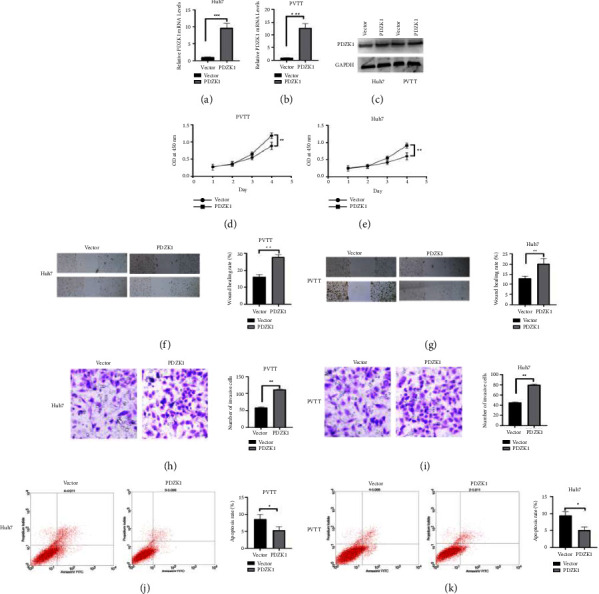
pcDNA3.1-PDZK1 affects the proliferation, migration, and apoptosis of HCC. (a, b, c) Western blot and RT-qPCR analysis showed that pcDNA3.1-PDZK1 strongly overexpression of PDZK1. (d, e, f, g, h, i) CCK8, wound healing, and transwell assay showed that pcDNA3.1-PDZK1 promote cell proliferation, invasion and migration. (j, k) Flow cytometry results revealed that pcDNA3.1-PDZK1 inhibit cell apoptosis. Data represent mean ± SD. ^*∗*^*P* < 0.05 compare with negative control.

Western blot and RT-qPCR analysis showed that pcDNA3.1-PDZK1 strongly over expression of PDZK1 (Figures 3(a)–3(c)), and the expression level increased significantly. CCK8, wound healing, and Transwell assay showed that pcDNA3.1- PDZK1 promote cell proliferation, invasion and migration (Figures 3(d)–3(i)), flow cytometry results revealed that sh-PDZK1 induced cell apoptosis (Figures 3(j) and 3(k)).

### 3.3. PDZK1 Affects HCC Function through STAT3/C-myc Pathway

**Figure 4:**
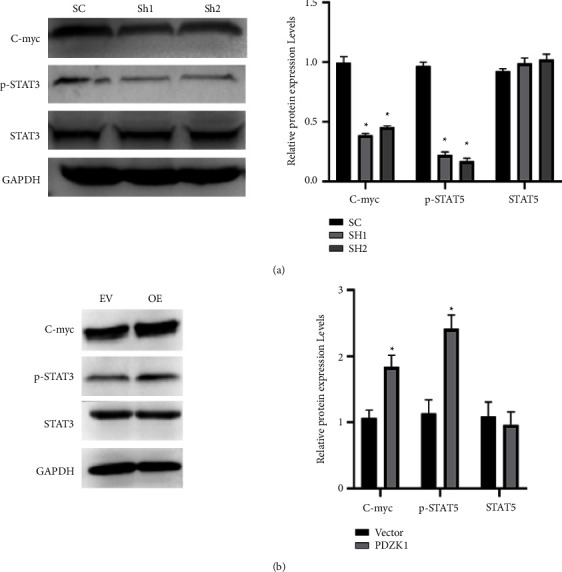
PDZK1 affects HCC function through STAT3/C-myc pathway. (a) Western blot results show that, compared with the control group, p-STAT3, C-myc protein expression significantly significantly decreased in the sh-PDZK1 group. (b) Western blot results show that, compared with the control group, p-STAT3, C-myc protein expression significantly increased in the pcDNA3.1-PDZK1 groups.

Uric acid may cause tumor immune response, so PDZK1 may also be related to tumor immune progress. The STAT3/C-myc pathway is closely related to tumor immune response. To investigated whether PDZK1 can affect liver cancer cell processes through the STAT3/C-myc pathway. Western blot results show that, compared with the control group, p-STAT3, C-myc protein expression significantly increased in the pcDNA3.1-PDZK1 groups, and the expression of these proteins were significantly decreased in the sh-PDZK1 group (Figures 4(a) and 4(b)).

### 3.4. Allopurinol Is an Effective Drug to Improve the Prognosis of HCC

**Figure 5:**
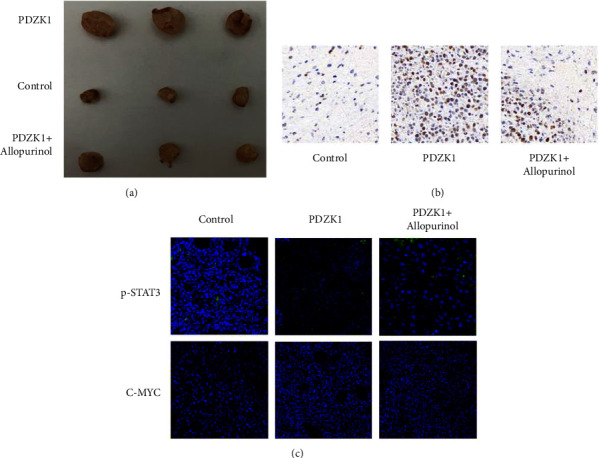
Allopurinol is an effective drug to improve the prognosis of HCC; (a) the tumor volume of PDZK1 overexpression group are increased, and treat with Allopurinol, the tumor volume is decreased; (b) Ki67 result showed that after treatment with Allopurinol, the proliferation of tumor was more reduced compare with PDZK1 overexpression group; (c) immunofluores result showed that,the p-STAT3 and C-myc expression was increased in PDZK1 overexpression group, and p-STAT3 and C-myc was decreased after treatment with allopurino.

We used high uric acid mice for tumorigenesis, 2*∗*10^6^ cells into mice with hyperuricemia. After subcutaneous transplantation of mice, allopurinol was fed every four days. The tumor volume of PDZK1 overexpression group are increased, and treat with allopurinol, the tumor volume is decreased (Figure 5(a)). Ki67 result showed that after treatment with allopurinol, the proliferation of tumor was more reduced compare with PDZK1 overexpression group (Figure 5(b)). Immunofluores result showed that, the p-STAT3 and C-myc expression was increased in PDZK1 overexpression group, and p-STAT3 and C-myc was decreased after treatment with allopurinol (Figure 5(c)).

### 3.5. PDZK1 Is Related to the Occurrence and Development of HCC in Clinically

**Figure 6:**
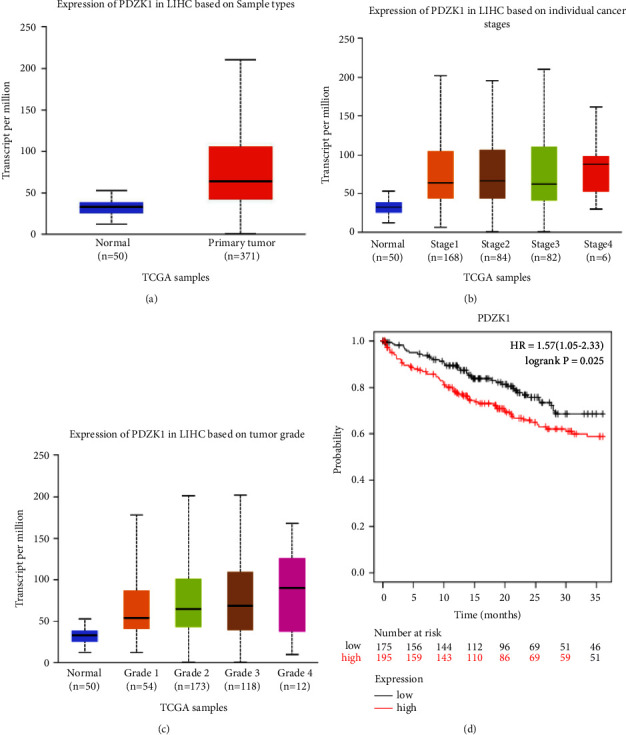
PDZK1 is related to the occurrence and development of HCC in clinically (a, b, c) the publicly online tools TGCA database, and showed that compared with normal, the expression of PDZK1 are increased in liver cancer, and increased in cancer stage (1, 2, 3, 4) and tumor grade (1, 2, 3, 4) (d) Kaplan–Meier Plotter results showed the prognosis of high expression of PDZK1 is poor in liver cancer.

We used the publicly online tools TGCA database, and showed that compared with normal, the expression of PDZK1 are increased in HCC, and increased in base individual cancer stage (1, 2, 3, and 4) and tumor grade (1, 2, 3, and 4) (Figures 6(a)–6(c)). Kaplan–Meier Plotter results showed the prognosis of high expression of PDZK1 is poor in HCC (Figure 6(d)).

## 4. Discussion

Hyperuricemia is a metabolic disease, which can be secondary to gout and increase the risk of other diseases, especially cardiovascular diseases, metabolic diseases, and kidney diseases, such as metabolic syndrome or heart failure [27, 28]. In recent years, through various clinical observations and studies, it has been found that hyperuricemia maybe an independent risk factor for a variety of solid tumors, including prostate cancer, colon cancer, and breast cancer [29–34]. In our study, the mRNA expression of PDZK1 was significantly increased after HCC after different concentrations of soluble uric acid treatment, which is similar to Chen et al. results [35]. The tumor volume of PDZK1 overexpression group are increased and after treat with allopurinol the tumor volume is decreased. Hyperuricemia may be related to the occurrence, development, and metastasis of malignant tumors.

With the deepening of research on the relationship between hyperuricemia and tumor, it is found that different immune cell subsets, cell surface receptors, and cytokines have significant effects on the pathogenesis of gout and tumor, and the relationship between hyperuricemia and tumor shows a complex trend. On the one hand, urate crystals can activate effective immune stimulants and trigger anticancer immune responses directly by reversing immunosuppression or as adjuvants. On the other hand, the interaction between urate crystals and immune cells can enhance immunosuppression and promote angiogenesis [36], thus, affecting the biological characteristics of malignant tumors.

Studies have shown that almost all PDZK1 proteins can combine with SLC17A1, ABCG2, URAT1, SLC17A3, and other transporters related to uric acid transport to form a complex regulating the absorption and excretion of uric acid [37], and act on the reabsorption and excretion of uric acid together. Our study shows that PDZK1 protein is highly expressed in HCC. Regulatory PDZK1 expression can affect the proliferation, migration, and apoptosis of HCC; uric acid may cause tumor immune response. It suggests that PDZK1 may be related to tumor immunity [38–40]. We detected the expression changes of STAT3/C-myc signal pathway protein after sh-PDZK1 or over expression of PDZK1. Knockdown of PDZK1 can reduce the expression of p-STAT3 and C-myc, Similar to previous results [41]. We also found that allopurinol maybe an effective drug to improve the prognosis of liver cancer.

## Data Availability

The basic data supporting our research results can be found through the e-mail of the corresponding author (qiuhongbin99@163.com).
